# Effect of Anisotropy of Reduced Graphene Oxide on Thermal and Electrical Properties in Silicon Carbide Matrix Composites

**DOI:** 10.3390/nano14060555

**Published:** 2024-03-21

**Authors:** Kamil Broniszewski, Jarosław Woźniak, Tomasz Cygan, Marek Kostecki, Dorota Moszczyńska, Marcin Chmielewski, Kamil Dydek, Andrzej Olszyna

**Affiliations:** 1 Faculty of Materials Science and Engineering, Warsaw University of Technology, ul. Wołoska 141, 02-507 Warsaw, Poland; kamil.broniszewski@pw.edu.pl (K.B.); jaroslaw.wozniak@pw.edu.pl (J.W.); tomasz.cygan@pw.edu.pl (T.C.); marek.kostecki@pw.edu.pl (M.K.); dorota.moszczynska@pw.edu.pl (D.M.); kamil.dydek@pw.edu.pl (K.D.); 2 Łukasiewicz Research Network, Institute of Microelectronics and Photonics, ul. Wólczyńska 133, 01-919 Warsaw, Poland; 3 Centre for Advanced Technologies, Adam Mickiewicz University in Poznan, 10 Uniwersytetu Poznanskiego St., 61-614 Poznan, Poland

**Keywords:** ceramics, composites, electrical properties, silicon carbide, reduced graphene oxide

## Abstract

Reduced graphene oxide, due to its structure, exhibits anisotropic properties, which are particularly evident in electrical and thermal conductivity. This study focuses on examining the influence of reduced graphene oxide in silicon carbide on these properties in directions perpendicular and parallel to the direction of the aligned rGO flakes in produced composites. Reduced graphene oxide is characterized by very high in-plane thermal and electrical conductivity. It was observed that the addition of rGO increases thermal conductivity from 64 W/mK (reference sample) up to 98 W/mK for a SiC–3 wt.% rGO composite in the direction parallel to the rGO flakes. In the perpendicular direction, the values were slightly lower, reaching up to 84 W/mK. The difference observed in electrical conductivity values is more significant and is 1–2 orders of magnitude higher for the flakes’ alignment direction. The measured electrical conductivity increased from 1.2710^−8^ S/m for the reference SiC sinter up to 1.55 × 10^−5^ S/m and 1.2410^−4^ S/m for the composites with 3 wt.% rGO for the perpendicular and parallel directions, respectively. This represents an enhancement of four orders of magnitude, with a clearly visible influence of the anisotropy of the rGO. The composite’s enhanced electrical and thermal conductivity make it particularly attractive for electronic devices and high-power applications.

## 1. Introduction

Silicon carbide is a material with a wide range of applications. The prevalence of strong covalent bonds contributes to the high hardness of this material but also to its low resistance to brittle fracture [1]. It exhibits high wear-resistance and is used as an abrasive material, in automotive applications for the production of bearings and brake discs, and in bulletproof vests [2,3]. It finds applications in high-temperature furnaces, nuclear reactors, and the electronics industry as a material for sensors and transistors. Silicon carbide is also employed in high-temperature heat exchangers [4,5,6]. It also demonstrates very high chemical resistance. Its exceptional resistance to oxidation is due to the formation of a thin layer of SiO_2_ on the grain surfaces.

Silicon carbide crystallizes in a hexagonal (α-SiC) or regular (β-SiC) system and exhibits polymorphism. It is a high-temperature semiconductor. In the case of silicon carbide monocrystal, thermal conductivity can reach up to 490 W/mK [7]. However, polycrystalline silicon carbide has a much lower thermal conductivity, not exceeding 170 W/mK. This is due to the presence of various defects in its microstructure, including pores, dislocations, impurities, and grain boundaries. In polycrystals, resistance and thermal conductivity depend on the sintering additives and the manufacturing process.

Silicon carbide is a hard to sinter material. This is attributed to the nature of its strong covalent bonds, a low self-diffusion coefficient, and a significant contribution of vaporization and condensation phenomena in the sintering process. Mass transport through the gas phase is unfavorable, leading to early inhibition of the sintering process and, consequently, only partial consolidation of the material [8]. Improved sinterability of SiC is achieved through the addition of sintering aids, typically in the form of oxides (Al_2_O_3_), their mixtures (Al_2_O_3_–Y_2_O_3_, Al_2_O_3_–AlN), or elements such as boron and carbon [9,10,11,12,13]. The key mechanism enhancing sinterability is the formation of a liquid phase. An exception is the addition of carbon, which is responsible for binding volatile silicon compounds formed during the thermal decomposition of silicon carbide and its vaporization during the sintering process. As a result, the unfavorable mass transport mechanism through the gas phase is reduced.

Graphene is a two-dimensional material with an extremely high surface area and remarkable mechanical, thermal, and electrical properties [14]. The thermal conductivity of suspended graphene ranges from 2000 to 5000 W/mK [15,16], and its Young’s Modulus is near to 1TPa. Graphene, owing to its structure, exhibits anisotropic properties. Due to its properties, graphene is a preferred material as a reinforcing phase in composites to enhance their electrical and thermal conductivities. It is used in electrodes (transparent conductive anodes for organic photovoltaic cells), sensors, lithium-ion batteries, and supercapacitors [17,18,19,20,21]. Additionally, graphene can increase the mechanical properties of composites, such as fracture-toughness or even tribological properties, and act as a solid lubricant [22,23]. This showcases the versatile role graphene can perform in modern industry. Due to the high demand for graphene, efforts are being made to obtain it using methods that would ensure high productivity at low costs. Such methods include a top-down approach with chemical reductions of graphene oxide, leading to the obtaining of graphene with a residual content of oxygen. Reduced graphene oxide (rGO) has inferior properties to pure graphene, but its higher availability and lower price are great advantages for industry. Due to the presence of oxygen-containing functional groups, rGO becomes more hydrophilic than pure graphene, enhancing the densification process during sintering and the rGO–matrix interface. Additionally, rGO is still characterized by high thermal and electrical properties, making it a focus for many researchers.

In composites containing graphene that are consolidated by methods where force is applied in a single axis during sintering (Spark Plasma Sintering, Hot Pressing), there is a phenomenon of graphene flakes aligning perpendicular to the axis of force application [24,25,26]. The same tendency is also observed for rGO. This leads to the development of anisotropy in the properties of such composites. This is particularly noticeable in the case of electrical and thermal conductivity. It is important to note that graphene, due to its highly developed surface, is also very prone to agglomeration due to Van der Waals forces [27]. This poses a significant challenge during the fabrication of composites involving this phase.

In recent years, graphene has been a primary focus for many researchers. Although there are numerous articles covering the anisotropy of thermal and electrical properties in graphene–ceramic matrix composites, those that specifically focus on reduced graphene oxide are scarce [28,29]. Furthermore, when considering the composite matrix material, only one research article was found. Hanzel et al. examined the anisotropy of thermal and electrical properties in SiC–rGO composites sintered using rapid hot pressing (RHP), in which the rGO was created as a result of the thermal reduction of GO during the sintering process [30]. In the last five years, only a few articles found in the Scopus database have focused on SiC–rGO composites. These cover interesting topics such as electromagnetic wave absorption [31], mechanical and tribological properties [32], or purely mechanical properties [33,34]. Unfortunately, none of them address the anisotropy of thermal or electrical properties. That scarcity of research on this topic was the authors’ motivation for writing this article.

## 2. Materials and Methods

Commercially available powders were used as substrates (Table 1). The composites were fabricated using powder metallurgy methods [35].

Eight powder compositions were prepared with a constant weight percentage of boron and varying weight percentages of rGO (Table 2). Boron was chosen as a sintering aid with its main purpose being to improve the sinterability of the SiC matrix composites through the formation of a liquid phase. Additionally, a reference sample was prepared with the same weight percentage of boron and the addition of carbon black to improve the sinterability of pure SiC. The role of the carbon black is solely as a sintering aid, and its influence on measured properties is minimal. The presence of boron and carbon black enables the fabrication of a high density compact without the need to increase the temperature or extend the sintering time. This is crucial to maintain consistency in phase composition and microstructure, ensuring the reliability of the material as a reference sample. The selection of these sintering aids aimed to sinter the silicon carbide sample under the same parameters as the other composites, closely resembling the properties of the pure SiC sample.

**Figure 1:**
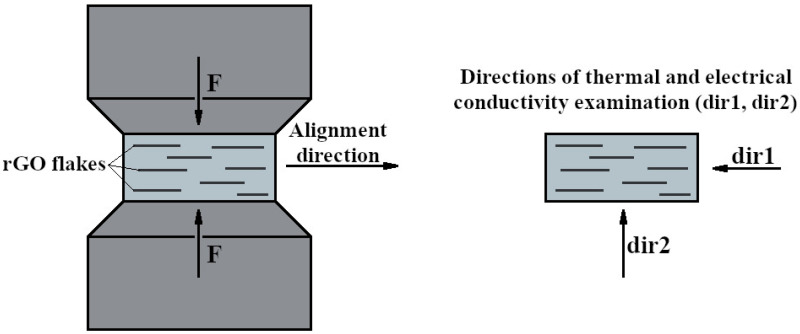
Direction of alignment of rGO flakes during sintering in SPS furnace, and directions of thermal and electrical properties examination.

The powder substrates were mixed in a ball mill (Fritsch Pulverisette) in propan-2-ol for 24 h, then dried at 70 °C and sieved (# = 250 μm). The densification of the SiC–rGO composites was carried out using the Spark Plasma Sintering (SPS) technique at 2000 °C for 1 h in an Argon atmosphere (HP-D10, FCT Systeme GmbH, Effelder-Rauenstein, Germany). The heating and cooling rate was set at 250 °C/min, and the pressure during sintering was maintained at 50 MPa. The SPS furnace operated in a DC pulse mode, configured to generate a 15 ms pulse with a 1 ms pause. The sintering parameters and pulse on/off ratio mentioned above are derived from the authors’ experience in sintering silicon carbide matrix composites. They were selected to ensure a high density of the composites with the shortest possible dwell time. Due to the anisotropic properties of the produced compacts, samples were cut in directions perpendicular and parallel to the axis of the force applied during the SPS sintering process. Figure 1 illustrates the schematic representation of the force direction and the measurement direction of the electrical and thermal properties. The relative density of the produced composites was measured using the Archimedes method. The theoretical density was determined using the mixture method. The phase composition of the composites was identified using X-ray diffraction (XRD) analysis (Bruker D8 Advance, Bruker Scientific Instruments, Billerica, MA, USA). Observations of the morphology of the powder substrates and the microstructure of the sinters were conducted using scanning electron microscopy (SEM, HITACHI S5500, Tokyo, Japan). Raman spectroscopy was carried out using a dispersive raman spectrometer with a 532 nm laser length (Nicolet Almega, Waltham, MA, USA, Thermo Scientific manufacturer). Electrical conductivity (Keithley A Tektronix Company 6221 Current Source 2182A Nanovoltmeter, Tektronix, Inc., Tokyo, Japan) and thermal conductivity (LFA 457, Netzsch, Selb, Germany) measurements were performed perpendicular and parallel to the direction of the force applied during the SPS sintering process.

## 3. Results

**Figure 2:**
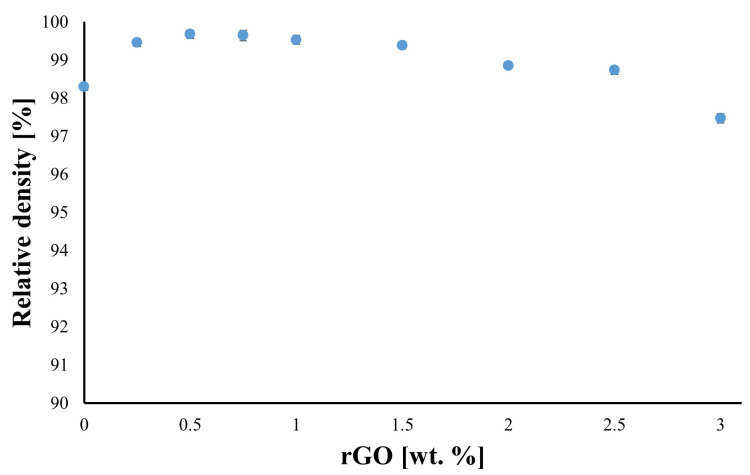
Relative density of sintered composites.

The silicon carbide-based composites exhibit high relative density, which diminishes with an increase in the rGO content, reaching the lowest value for the SiC–3 wt.% rGO composite (97.5%) (Figure 2). Due to the large decrease in density for this composite, it was decided to limit the maximum rGO addition for the manufactured composites to 3 wt.%. Further increases in the amount of rGO, and thus the porosity of the composites, could compromise the reliability of the results. Excessive porosity significantly influences the results, leading to distortion and making correct interpretation difficult or impossible. A maximum is observed for the obtained density values, occurring in the composite with 0.5% weight rGO content. Only when the rGO content exceeds 2% is a decrease in relative density below 99% observed.

**Figure 3:**
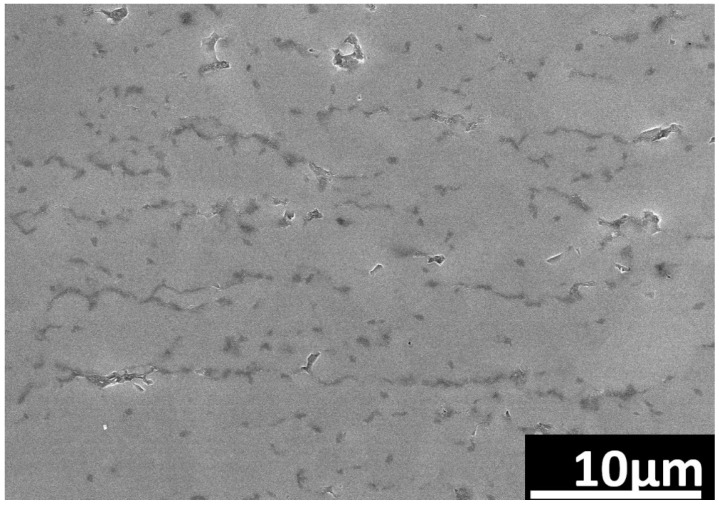
SEM image of rGO parallel alignment in SiC–2 wt.% rGO composite.

**Figure 4:**
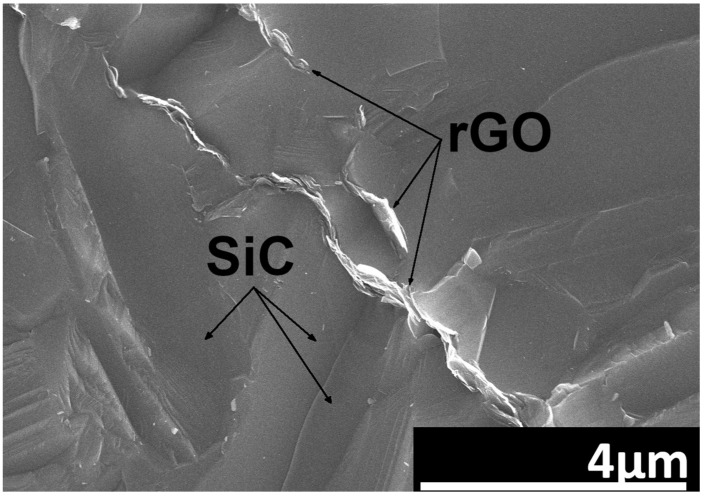
The fracture surface of SiC–2 wt.% rGO composite.

**Figure 5:**
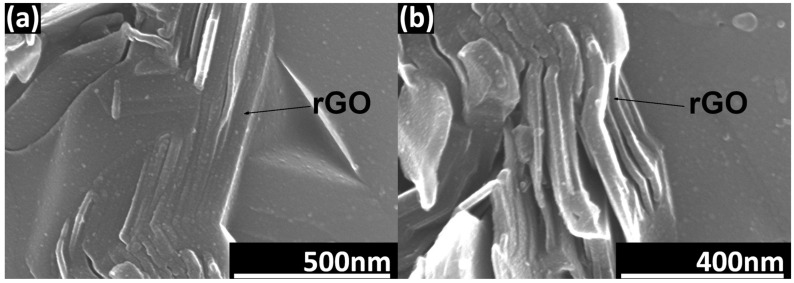
SEM image of SiC–rGO interface. (**a**) No visible porosity between rGO flakes; (**b**) significant porosity between rGO flakes can be observed.

**Figure 6:**
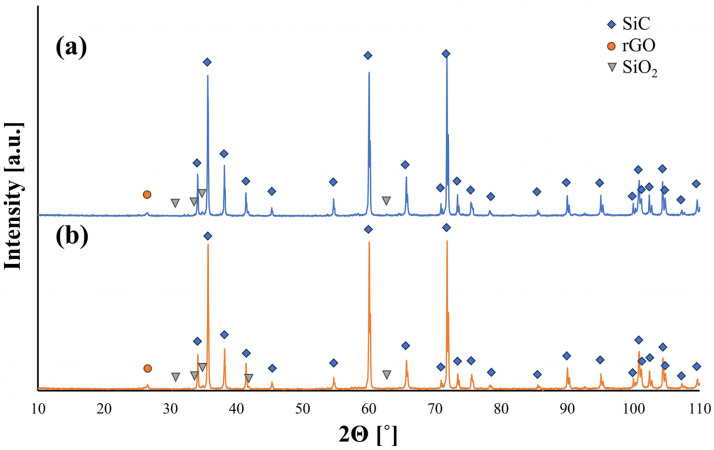
XRD patterns of (**a**) SiC–2 wt.% rGO composite; (**b**) SiC–3 wt.% rGO composite.

A tendency of the rGO flakes to align parallel in one direction is observed (Figure 3). This direction coincides with the direction perpendicular to the axis of the force applied during the SPS sintering process. Microstructure observations reveal the presence of elongated grains (Figure 4). Observations conducted with scanning electron microscopy indicate a strong interface between rGO and the silicon carbide matrix. Reduced graphene oxide tends to form agglomerates containing pores, which negatively impact mechanical, electrical, and thermal properties. Loosely connected rGO flakes can be observed, but there are no pores at the SiC–rGO boundary (Figure 5). The phase analysis results, conducted on two representative composites, indicate that the obtained sinters consist of three phases: SiC (6H), rGO, and trace amounts of SiO_2_ (Figure 6).

**Figure 7:**
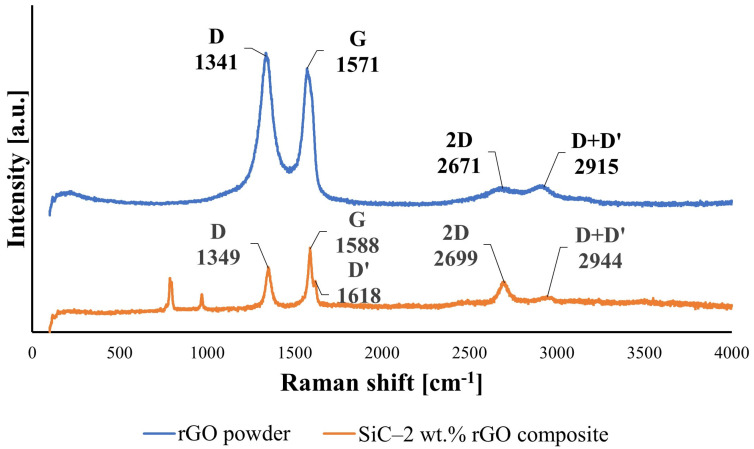
Raman spectra of rGO powder substrate and SiC–2 wt.% rGO composite.

Raman spectroscopy showed a series of bands for the rGO powder substrate and the exemplary composite with 2 wt%. rGO that are characteristic for carbon structures (Figure 7). The D band and G band for the rGO powder are at the positions of 1341 cm^−1^ and 1571 cm^−1^. For the SiC–2 wt.% rGO composite, we can observe a shift in these bands’ positions to 1349 cm^−1^ and 1588 cm^−1^. Additionally, a broad 2D band was observed with a peak at 2671 cm^−1^ and D + D′ band at 2915 cm^−1^ for the rGO substrate and with slightly shifted positions to 2699 cm^−1^ and 2944 cm^−1^ for the produced composite. It should be noted that, for the produced composites, a D’ band at position 1618 cm^−1^ can also be observed. The calculated ID/IG ratio for the pure rGO powder and SiC–2 wt.% rGO sinter equals to 1.11 and 0.77, respectively.

**Figure 8:**
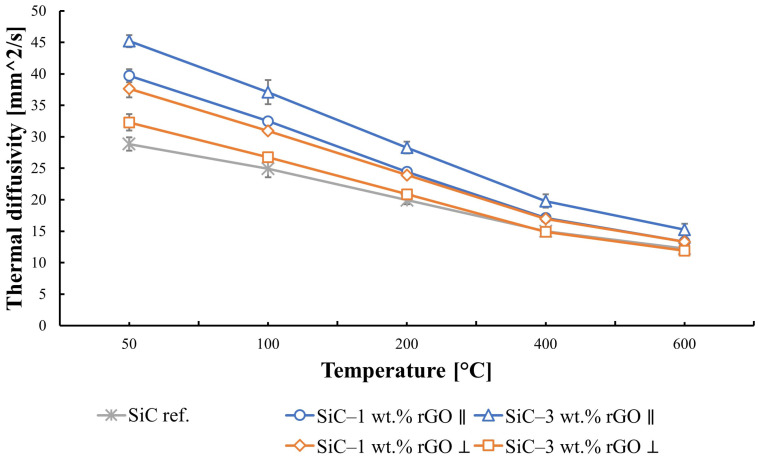
Thermal diffusivity of produced composites in both directions: parallel and perpendicular to the graphene platelets.

The obtained results for thermal diffusivity and thermal conductivity are highly dependent on the direction in which they were measured. An influence of the anisotropy of the produced composites on these properties is observed. Thermal diffusivity increases with the rGO content in the SiC matrix for the direction parallel to the rGO flakes, reaching the highest value for the composite with 3 wt.% rGO (Figure 8). At a temperature of 50 °C, it equals to 45 mm^2^/s. The reference sample value at this temperature is 29 mm^2^/s. It was observed that, for the direction perpendicular to the reduced graphene oxide flakes, the highest value is reached by a composite with 1 wt.% rGO, and it equals to 37 mm^2^/s. With an increase in temperature, the thermal diffusivity of the produced composites decreases, dropping below 16 mm^2^/s for the maximum measurement temperature of 600 °C.

**Figure 9:**
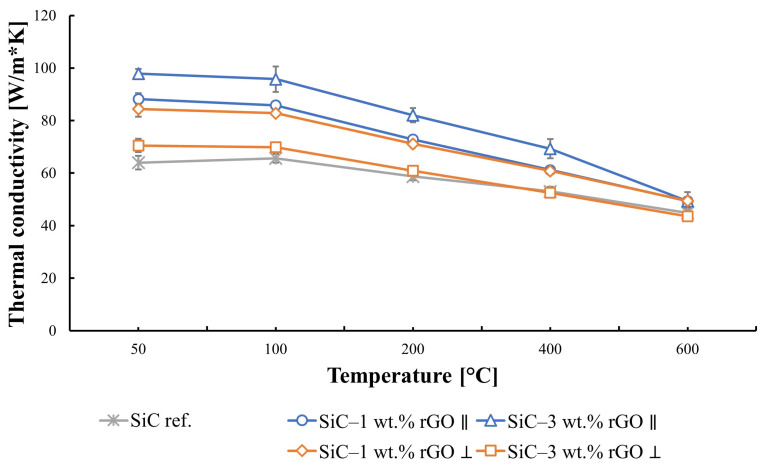
Thermal conductivity of produced composites in both directions: parallel and perpendicular to the graphene platelets.

In the case of thermal conductivity, a similar trend can be observed (Figure 9). The addition of rGO increases the thermal conductivity from 64 W/mK for the reference sample up to 98 W/mK for the composite containing 3 wt.% rGO in the parallel direction. In the perpendicular direction, it reaches 84 W/mK for the SiC–1 wt.% rGO composite. Raising the measurement temperature reduces thermal conductivity, which reaches its minimum value below 50 W/mK at 600 °C.

**Figure 10:**
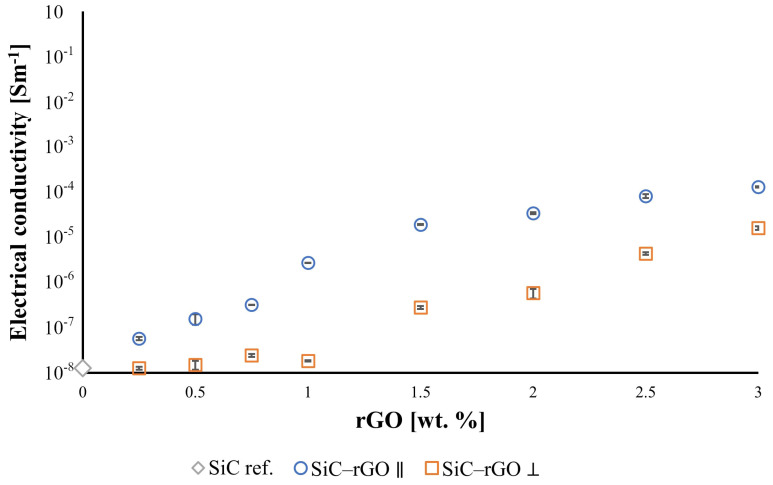
Electrical conductivity of produced composites in both directions: parallel and perpendicular to the graphene platelets.

The addition of rGO to the SiC matrix provides a noticeable increase in the electrical conductivity of the composites compared to the reference sample (1.27 × 10^−8^ S/m) (Figure 10). Increasing the reduced graphene oxide content in the silicon carbide matrix leads to an increase in electrical conductivity for the sample with the highest rGO content (3 wt.%) by four orders of magnitude to 1.24 × 10^−4^ S/m in the direction parallel to the alignment of rGO flakes. In the perpendicular direction, the measured value was one order of magnitude lower (1.55 × 10^−5^ S/m). It was observed that electrical conductivity measured in the direction of the rGO alignment is always one or two orders of magnitude higher, reflecting the anisotropy of the properties of the produced composites.

## 4. Discussion

The addition of reduced graphene oxide leads to an increase in the density of the produced composites, reaching a maximum for the sinter containing a small fraction of rGO. This trend has been observed for SiC–Graphene composites in other studies [36]. The highest density is in the region of low rGO content, not exceeding 1% weight. Beyond the critical rGO content, there is a constant decrease in the density of the composites (unimodal character). This is because silicon carbide, as a refractory material, requires the addition of sintering aids, in this case boron and carbon. Boron is supplied in the form of amorphous powder, and rGO serves as the carbon source. At low concentrations, carbon acts as a sintering activator, contributing to the increased density of the compacts. It is essential to note that rGO also tends to form agglomerates, which negatively impact the sinters’ densification. The combination of these two processes results in the observed density maximum in the relationship between density and rGO content in the composites. Reduced graphene oxide concentrations exceeding the critical value only lead to a further decrease in density due to the continuously growing number of rGO agglomerates. However, this does not imply that the thermal and electrical properties of the composites reach their maximum at the same point of maximum density.

**Figure 11:**
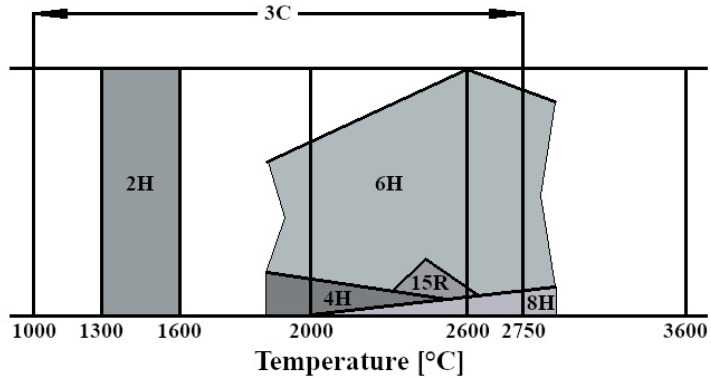
Diagram of thermodynamic stability of SiC [8].

X-ray diffraction results confirmed that a phase transformation of silicon carbide occurred during the consolidation process, shifting from a regular to a hexagonal structure with a 6H polytype. Additionally, the presence of elongated grains, characteristic of the hexagonal phase, indicates this phase transformation. Ragaru et al. observed similar phase transformations in composites containing carbon and boron [37]. Zhou et al. also noticed a tendency for phase transformation in silicon carbide sintered using the Pulse Electric Current Sintering (PECS) method [38]. The hexagonal variant of silicon carbide is referred to as the α-SiC and is a stable phase. The thermodynamic stability range for silicon carbide shows that, at a sintering temperature of 2000 °C, three polytypes of the hexagonal phase are possible: 6H, 4H, and 8H. The occurrence of the 8H polytype is negligible at this temperature (Figure 11). The peak at 2θ = 26.5 was identified as the main peak of rGO indexed to the (002) planes, which is similar to that reported by other researchers [39,40]. It can also be observed that with a higher content of reduced graphene oxide in the composite, the peak from rGO is characterized by a higher intensity, which correlates with theoretical knowledge.

Raman spectra of the rGO powder substrate revealed D and G bands at positions of 1341 cm^−1^ and 1571 cm^−1^, respectively. The measured bands positions for the produced composite were slightly shifted into the direction of higher values, to 1349 cm^−1^ and 1588 cm^−1^. The D-band is associated with the number of defects and is visible only when there is disorder in the structure, while the G-band is an effect of C–C stretching and appears in all carbon structures. A broad 2D band is also observed with peaks at 2671 cm^−1^ and 2915 cm^−1^ for rGO powder, and 2699 cm^−1^ and 2944 cm^−1^ for the composite. The 2D band is very sensitive to stacking graphene layers. The peaks for the composite are slightly shifted towards higher values, implying that the stacking of rGO occurred, and that the produced composites are reinforced with multi-layered rGO. This, along with XRD results, confirms that the sintering parameters of the SPS technique did not cause graphitization, and the reduced graphene oxide is still present in the produced composites. The I_D_/I_G_ ratio is a parameter related to the disorder within the material and is influenced by crystallite size. It has been noted that, for a crystallite size below 2 nm, the disorder increases with the decrease in I_D_/I_G_ ratio [41]. This work presents results for rGO flakes in the range of micrometers, and, thus, the higher the I_D_/I_G_ ratio, the higher the disorder of material. The I_D_/I_G_ measured value equals to 1.11 and 0.77 for the rGO substrate and SiC–2 wt.% rGO composite, respectively. It can be seen that the I_D_/I_G_ parameter decreased after consolidation by the SPS method quite significantly. Additionally, the presence of the D’ band was observed in the sintered composite, which is also correlated with the level of disorder. The more defected the graphene is, the stronger the D’ band becomes, and it also shifts to the lower values of frequencies [42,43]. In the rGO powder substrate, it cannot be seen because it completely overlaps with the G band. If we take into consideration the position of the D + D′ band we can calculate that the peak of the D’ band would equal to 1574 cm^−1^, which is indeed very close to the peak of G band, making it impossible to tell them apart. The visible lower number of defects may be related to further reduction of rGO during sintering at 2000 °C. Similar behavior was reported during the reduction of graphene oxide that occurred in composites produced by the HPHT (High Pressure High Temperature) method [44]. The decrease in residual oxygen would explain the values of I_D_/I_G_ ratio and appearance of the D’ band.

The thermal conductivity of silicon carbide, according to the literature, falls within the range of 45 to 170 [W/mK] [45]. The obtained results for the reference sample are 64 [W/mK]. Due to the tendency of rGO flakes to align along the direction perpendicular to the axis of applied force during the consolidation process, the results obtained in the two directions differ significantly. Anisotropy is noticeable and results from the very high in-plane thermal conductivity of rGO, reaching values above 2000 W/mK. The increase in thermal conductivity caused by adding rGO to the silicon carbide matrix is not linear. The addition of 1% weight rGO led to an increase in thermal conductivity of 24 W/mK, compared to the reference sample. At the same time, the addition of 3 wt.% of rGO to the composite matrix resulted in an increase in thermal conductivity of 37 W/mK. The impact of reduced graphene oxide flake agglomeration is visible here, leading to increased porosity in the produced compacts and, as a result, lower thermal conductivity. A sudden decrease in thermal conductivity for the SiC–3 wt.% rGO composite measured in the direction perpendicular to the alignment of rGO flakes is observed. In this case, the influence of porosity on thermal conductivity outweighs the impact of adding a higher amount of rGO. With the increase in temperature, diffusivity and thermal conductivity decrease, which aligns with the theoretical expectations for dielectric material [45]. Thermal conductivity mainly depends on the propagation of phonons. At an elevated temperature, phonon scattering becomes a greater factor, due to phonon–phonon interaction and increased crystal lattice vibration. This leads to a decrease in the mean free path of phonons and, ultimately, thermal conductivity.

The electrical conductivity for silicon carbide depends on many parameters, such as sintering atmosphere, additives, and the presence of metal nitrides. Therefore, electrical conductivity values lie in a very wide range of 10^−3^ to 10^−11^ S/m [46,47,48]. The not-doped silicon carbide electrical conductivity is closer to the lower values of this spectrum, which aligns with the measured value for the reference SiC sinter (1.2710^−8^ S/m). Even a small addition of reduced graphene oxide to the silicon carbide matrix causes a significant improvement in the electrical conductivity of the produced composites. Its rapid increase is particularly evident for compacts containing up to 1.5% weight rGO. These results correlate well with the obtained density values, which noticeably decrease for composites containing more than 1.5% weight rGO. Nevertheless, even the decrease in density observed at higher reduced graphene oxide contents did not neutralize its significant influence on the increase in the electrical conductivity of the tested samples. This is due to the very high electrical conductivity of rGO itself, which is 6 × 10^3^ S/m. Hence, the highest electrical conductivity was observed in the parallel direction for the composite with the highest rGO content (1.24 × 10^−4^ S/m). Similarly to thermal conductivity, there is a significant impact of anisotropy on electrical conductivity.

## 5. Conclusions

The consolidation of SiC–rGO composites using the SPS technique results in obtaining sinters characterized by a relative density of over 99% for a rGO content below 2 wt.%. The lowest value of 97.5% was measured for the SiC–3 wt.% rGO composite. Scanning electron microscopy revealed a tendency of reduced graphene oxide to create agglomerates containing pores, negatively impacting the properties of the produced composites. It is worth noting that no pores were observed at the boundary of the rGO flakes and SiC matrix, indicating a strong interface between these phases. SEM observations also confirmed the alignment of rGO in the direction perpendicular to the axis of force application during SPS sintering. The produced sinters consist of three phases: SiC (6H), rGO, and trace amounts of SiO_2_. X-ray diffraction showed the presence of a hexagonal silicon carbide, implying that, during sintering, the phase transformation of β-SiC into α-SiC occurred. This was confirmed by SEM images showing elongated SiC grains. Raman spectroscopy confirmed the presence of reduced graphene oxide in the produced composites. The appearance of the D’ band and a decrease in the value of I_D_/I_G_ ratio in composites to 0.77 indicate that, during sintering, a further reduction of rGO occurred. The addition of rGO enhanced both thermal diffusivity and conductivity. The obtained values are highly dependent on the direction in which they were measured, and they are the highest for the SiC–3 wt.% rGO sinter in the direction parallel to the rGO flakes’ alignment. These equal to 45 mm^2^/s and 98 W/mK for thermal diffusivity and conductivity, respectively. An increase in temperature leads to a shorter mean free path of phonons and ultimately to a decrease in thermal diffusivity and conductivity. A very strong influence of anisotropy was observed in the measured electrical conductivity values. In the perpendicular direction, the measured electrical conductivity was always at least one order of magnitude lower than in the parallel direction. The highest value was measured for the composite with the highest content of rGO and equals to 1.24 × 10^−4^ S/m. This means that the addition of 3 wt.% rGO leads to a four-order-of-magnitude increase compared to the reference SiC sinter (1.2710^−8^ S/m).

## Figures and Tables

**Table 1:** Powder substrates used for producing composites.

Powder	APS ^1^	Manufacturer
Silicon carbide	0.42 µm	Alfa Aesar (Ward Hill, MA, USA)
rGO	<40 µm	Łukasiewicz Research Network (Warsaw, Poland)
Boron	0.39 µm	International Enzymes Limited (Fareham, UK)
Carbon	<100 nm	Sigma-Aldrich (Burlington, VT, USA)

^1^ Average particle size.

**Table 2:** Composition of produced SiC matrix composites.

Composites								
Boron [wt.%]	0.3	0.3	0.3	0.3	0.3	0.3	0.3	0.3
rGO [wt.%]	0.25	0.5	0.75	1	1.5	2	2.5	3
Reference sample								
Boron [wt.%]			0.3					
Carbon black [wt.%]			0.5					

## Data Availability

All data included in this study are available upon request by contact with the corresponding author.
